# Cognitive impairment and p‐tau217 are high in a vascular patient cohort

**DOI:** 10.1002/alz.70565

**Published:** 2025-08-07

**Authors:** Scott R. French, Juan C. Arias, Summan Zahra, Madeline Ally, Cris Escareno, Emma Heitkamp, Franchell Vazquez, Madison Hillis, Haley Wiskoski, Karthik Ainapurapu, Gavin Culwell, Caronae Howell, Kevin Johnson, Cody Kraemer, John Pacanowski, Luis Leon, Scott Berman, Federico Yanquez, Joshua Balderman, Joseph Sabat, Olivia Hung, Layla Lucas, Francesca Vitali, Edward J. Bedrick, Raza Mushtaq, Maria Altbach, Theodore P. Trouard, Fanny M. Elahi, Nicholas J. Ashton, Jeffrey L. Dage, Eric M. Reiman, Gene E. Alexander, Craig C. Weinkauf

**Affiliations:** ^1^ The Division of Vascular Surgery University of Arizona Tucson Arizona USA; ^2^ Department of Psychology University of Arizona Tucson Arizona USA; ^3^ Department of Medical Imaging University of Arizona Tucson Arizona USA; ^4^ Pima Heart and Vascular Tucson Arizona USA; ^5^ Department of Cardiovascular Medicine, Sarver Heart Center University of Arizona College of Medicine Tucson Arizona USA; ^6^ Department of Neurology University of Arizona Tucson Arizona USA; ^7^ Center for Innovation in Brain Science University of Arizona Tucson Arizona USA; ^8^ Department of Epidemiology and Biostatistics, Mel and Enid Zuckerman College of Public Health University of Arizona Health Sciences Center Tucson Arizona USA; ^9^ Department of Neuroradiology Barrow Neurological Institute Phoenix Arizona USA; ^10^ Department of Biomedical Engineering University of Arizona Tucson Arizona USA; ^11^ BIO5 Research Institute University of Arizona Tucson Arizona USA; ^12^ Evelyn F. McKnight Brain Institute University of Arizona Tucson Arizona USA; ^13^ Department of Neurology and Department of Neuroscience Icahn School of Medicine at Mount Sinai New York New York USA; ^14^ Department of Psychiatry and Neurochemistry, Institute of Neuroscience & Physiology the Sahlgrenska Academy at the University of Gothenburg Göteborg Sweden; ^15^ Banner Sun Health Research Institute Sun City Arizona USA; ^16^ Banner Alzheimer's Institute Phoenix Arizona USA; ^17^ Department of Neurology Indiana University School of Medicine Indianapolis Indiana USA; ^18^ Evelyn F. McKnight Brain Institute, Department of Psychiatry, Neuroscience and Physiological Sciences Graduate Interdisciplinary Programs and BIO5 Institute University of Arizona and Arizona Alzheimer's Disease Consortium Tucson Arizona USA

**Keywords:** Alzheimer's disease, blood biomarkers, cognitive impairment, vascular disease, vascular risk factors

## Abstract

**INTRODUCTION:**

Vascular comorbidities are modifiable contributors to cognitive impairment and Alzheimer's disease (AD), yet brain health outcomes are rarely evaluated in cardiovascular patients.

**METHODS:**

This study prospectively evaluated cognition and AD pathology in 162 community‐dwelling adults with asymptomatic cardiovascular disease who did not have a clinical diagnosis of dementia or cognitive impairment.

**RESULTS:**

Twenty‐nine percent of the cohort had Montreal Cognitive Assessment (MoCA) scores indicative of cognitive impairment or dementia after adjusting for age, sex, and education based on National Alzheimer's Coordinating Center normative data. AD blood biomarker phosphorylated tau217 was elevated in 55% of the cohort, significantly associated with decreased MoCA scores (*β* = −1.46, 95% confidence interval [CI] −2.53 to −0.39, *p* < 0.01), and accurately differentiated cognitive impairment (area under the curve 0.94, 95% CI 0.88–0.99).

**DISCUSSION:**

This level of undiagnosed cognitive impairment and AD pathology exceeds what would be expected in the general population and highlights a potential need for screening and future work to better identify treatment options.

**Highlights:**

- Brain health outcomes are rarely evaluated in vascular patients.
- One hundred sixty‐two adults with asymptomatic cardiovascular disease but without diagnoses of cognitive impairment or dementia were evaluated.
- Phosphorylated tau217 accurately differentiated cognitive impairment in patients with cardiovascular disease.
- High levels of cognitive impairment and Alzheimer's disease pathology are greatly underdiagnosed in the cardiovascular population.

## 1 BACKGROUND

Vascular comorbidities are increasingly recognized as contributors to cognitive impairment and Alzheimer's disease (AD) risk; however, vascular patients are not routinely screened for cognitive dysfunction. With the rising life expectancy, the prevalence of AD is projected to triple by 2050.^1^ In the United States, an estimated 92% of mild cognitive impairment (MCI)^2^ and 39% of AD^3^ cases remain undiagnosed. AD blood‐based biomarkers are expected to narrow this gap, offering opportunities for early diagnosis and implementation of strategies to reduce AD risk. The expanded use of AD blood‐based biomarkers is particularly compelling in the wake of emerging AD disease‐modifying pharmacological therapies.^4^

Unlike non‐modifiable AD risk factors such as age, sex, and apolipoprotein E (*APOE*) ε4 allele, vascular diseases are modifiable through lifestyle, pharmacological, and surgical interventions, and treating them can reduce AD risk.^5^, ^6^ Although these and other data showing that midlife vascular risk factors such as smoking, hyperlipidemia, diabetes, and hypertension increase risk for AD^7^, ^8^, ^9^, ^10^, ^11^, ^12^, ^13^ highlight the relevance of vascular diseases in AD pathogenesis, cognitive function and AD risk often remain overlooked in major cardiovascular clinical trials, including the Justification for the Use of Statins in Primary Prevention: An Intervention Trial Evaluating Rosuvasatin^14^ and Diabetes Prevention Program^15^ studies. Due to limited work exploring cognitive dysfunction in vascular populations, the American Heart Association and Society for Vascular Surgery guidelines for the management of vascular diseases do not currently recommend screening for cognitive/AD outcomes.^16^, ^17^, ^18^ Brain‐health assessments are rarely integrated into the clinical evaluations of vascular patients, contributing to the underdiagnosis of cognitive impairment in this population.

Current clinical AD risk assessment relies on extensive cognitive and behavioral testing^19^ and positron emission tomography (PET)/cerebrospinal fluid (CSF) biomarkers of amyloid beta (Aβ) and tau; however, they have not been widely adopted in clinical settings due to cost and limited availability. Blood‐based biomarkers of AD pathology are increasingly accurate tools to quantify AD risk, demonstrating abnormal levels years before the onset of cognitive changes.^20^, ^21^ Of the candidate AD blood‐based biomarkers, phosphorylated tau (p‐tau)217 has emerged as most promising for clinical translation, showing high diagnostic accuracy rivaling that of PET and CSF biomarkers in its ability to detect AD pathology.^22^, ^23^, ^24^, ^25^ Anastasi et al. compared the performance of plasma biomarkers in detecting AD, finding that p‐tau217, regardless of the assay, predicted AD with the highest accuracy.^24^ In another study, p‐tau217 demonstrated high accuracy in predicting Aβ and tau positivity, regardless of whether CSF or PET imaging was used to define positivity.^22^ Furthermore, p‐tau217 outperformed all other plasma biomarkers in predicting AD pathology and cognitive decline,^22^, ^23^ and strongly correlated with amyloid and tau PET, Braak stages, and neuropathologically defined AD.^22^, ^25^

As clinicians regularly seeing patients for longitudinal care of their cardiovascular diseases, we suspected that there were high levels of undiagnosed cognitive impairment and AD risk in our patient population. In agreement with these clinical observations, there is growing recognition that cardiovascular patients may have a disproportionately greater risk for the development of AD.^7^, ^8^, ^9^, ^10^, ^11^, ^12^, ^13^ However, data evaluating early diminished cognitive function and AD pathology in vascular cohorts have been limited, representing a potential critical gap in both research and clinical care for a relatively large swath of the aging population. The potential gap in clinical care is further reflected by the lack of cardiovascular society guidelines addressing brain health outcomes in this population. Understanding the prevalence of cognitive impairment and AD pathology in cardiovascular populations has multidisciplinary implications for the clinicians (neurologists, cardiologists, primary care doctors, and vascular surgeons) who care for these patients in the community, and for researchers in the dementia field who may recognize a gap in basic science knowledge and the potential for targeted therapies for this population. As such, we sought to test our hypothesis that community‐dwelling patients with vascular comorbidities (who are regularly seen by physicians) have high rates of undiagnosed cognitive impairment and AD pathology. We evaluated cognitive function using the Montreal Cognitive Assessment (MoCA) and AD pathology using plasma biomarkers of p‐tau217 and Aβ42/40, thereby selecting brain health measures that could be more readily translated to cardiovascular clinical settings.

**RESEARCH IN CONTEXT**

1. **Systematic review**: We reviewed the literature using traditional online databases (e.g., PubMed). Evidence indicates vascular comorbidities are modifiable risk factors for Alzheimer's disease (AD). However, the prevalence of cognitive impairment and AD pathology in cardiovascular populations is not well defined, and clinical guidelines for the management of vascular patients do not recommend screening for cognitive outcomes in the vascular patient population.
2. **Interpretation**: One hundred sixty‐two community‐dwelling adults with asymptomatic cardiovascular disease and without prior diagnoses of cognitive impairment and dementia were evaluated. Twenty‐nine percent of the cohort had Montreal Cognitive Assessment scores indicative of cognitive impairment or dementia, and 55% had elevated plasma phosphorylated tau (p‐tau)217. This degree of cognitive impairment and AD pathology far exceeds what would be expected in the general population.
3. **Future directions**: Our study raises the possibility of screening for brain health outcomes in cardiovascular populations. In addition, our findings suggest p‐tau217 may be a valid tool for screening this population. Additional studies are warranted to validate our findings and better identify early treatment options for this population.

## 2 METHODS

### 2.1 Study cohort

Participants enrolled in the prospective Carotids and Mind (CAM) clinical study were used for this analysis. CAM is a longitudinal observational study that evaluates the effect of asymptomatic extracranial carotid atherosclerotic disease (aECAD) and other vascular risk factors and diseases on cognitive impairment and AD risk. In this analysis, 162 adults between the ages of 50 and 85 were prospectively recruited from vascular surgery or cardiology academic and community health clinics in Tucson, Arizona, from 2022 to 2024. Individuals were being evaluated in clinic for vascular risk factor management (i.e., blood pressure control), asymptomatic cardiac disease, asymptomatic carotid disease, or other chronic vascular diseases. Individuals were eligible to participate if they had two or more vascular risk factors or diseases, including smoking (current smoker or ≥ 10 pack‐year history), hypertension, hyperlipidemia, diabetes mellitus, coronary artery disease, or peripheral arterial disease and/or a clinical diagnosis of aECAD. Participants were excluded from these analyses if they had a prior clinical diagnosis of dementia, MCI, major depression, neurological disorders, end‐stage renal disease, heart failure, or terminal cancer. Patients with symptomatic cerebrovascular events (stroke or transient ischemic attack within the previous 6 months) were also excluded. We use the word *asymptomatic* to describe this cohort, reflecting their clinical cerebrovascular and cardiac status. Demographic information and past medical history were determined based on participants’ self‐report (reviewed by study coordinators with participants) and medical records review. All study procedures were approved by the institutional review board at the University of Arizona. All participants gave written informed consent after demonstrating an understanding of the study procedures.

### 2.2 Blood‐based biomarkers and *APOE* genotyping

Blood was drawn using lithium heparin vacutainer tubes and immediately transported to the University of Arizona Biorepository for processing and storage at −80°C. Plasma was collected by centrifugation at 1200 × g for 10 minutes at 25°C. Peripheral blood mononuclear cells (PBMCs) were isolated according to published protocols.^26^ Samples underwent one freeze–thaw cycle prior to analysis. Plasma p‐tau217, Aβ42/40, and *APOE* genotypes were quantified through collaboration with the Biomarker Assay Laboratory at the National Centralized Repository for Alzheimer's Disease (NCRAD), which performs biomarker testing for several large studies.^27^, ^28^, ^29^, ^30^, ^31^

Plasma Aβ42 and Aβ40 were measured using the Quanterix Simoa N4PE HD‐X Advantage Kit (Quanterix, Cat. 103670, Lot. 503864). The average Aβ42 and Aβ40 concentrations obtained from duplicate wells were used to calculate the Aβ42/40 ratio, which was used for the analysis. Plasma p‐tau217 was measured using the Quanterix Simoa ALZpath pTau217 HD‐X Advantage Kit (Quanterix, Cat. 104371, Lot. 999024). The average p‐tau217 concentrations obtained from duplicate wells were used for the analysis. Length of storage at −80°C prior to performing the assay did not affect plasma p‐tau217 or Aβ42/40 levels (Figure S1 in supporting information). Plasma p‐tau217 and Aβ42/40 were binarized according to previously published cutoffs of PET positivity for subsequent analyses; a cutoff of > 0.42 pg/mL was used to define abnormal plasma p‐tau217 and < 0.045 was used to define abnormal plasma Aβ42/40.^22^, ^32^ *APOE* gene variants (ε2, ε3, ε4) were defined within our cohort by analysis of single nucleotide polymorphisms at rs429358 and rs7412. *APOE* data were binarized according to ε4 carriership, including ε2/ε4 and ε3/ε4 genotypes for all analyses. Exclusion of *N* = 3 *APOE* ε2/ε4 participants did not change the obtained results. Six participants were missing plasma biomarkers and *APOE* genotypes and were excluded from the biomarker analyses.

### 2.3 Neurocognitive testing

Study participants underwent neurocognitive testing using the MoCA,^33^ following National Alzheimer's Coordinating Center (NACC) protocols. Initial scoring was conducted under the supervision of a neuropsychologist blinded to biomarker findings (G.E.A.). Raw MoCA scores were transformed into *z* scores adjusting for age, sex, and education level using the NACC Uniform Data Set (UDS) version 3.0 norms calculator (https://neuropsychdata.com/normscalculator/).

### 2.4 Statistical analysis

#### 2.4.1 Primary analysis

Frequency plots and pie charts were generated to visualize the overall distribution of NACC‐adjusted MoCA scores and plasma biomarker levels within the vascular cohort. To determine associations of plasma biomarkers with raw MoCA scores, we performed a multivariate linear regression accounting for age (in years), sex, race and ethnicity, education years, and *APOE* ε4 carriership. Discriminative ability of p‐tau217 for cognitive status was evaluated using receiver operating characteristic (ROC) curves.

#### 2.4.2 Sensitivity analyses

Due to the known contributions of stroke and chronic kidney disease (CKD) to our outcomes of interest,^34^, ^35^ we generated additional frequency plots and performed additional multivariate linear regressions to address these potential confounders.

A *p* value < 0.05 was considered statistically significant. Statistical analysis was conducted using R Studio (2023.12.1+402) by S.R.F., J.C.A., and S.Z.

## 3 RESULTS

### 3.1 Primary analysis

**FIGURE 1:**
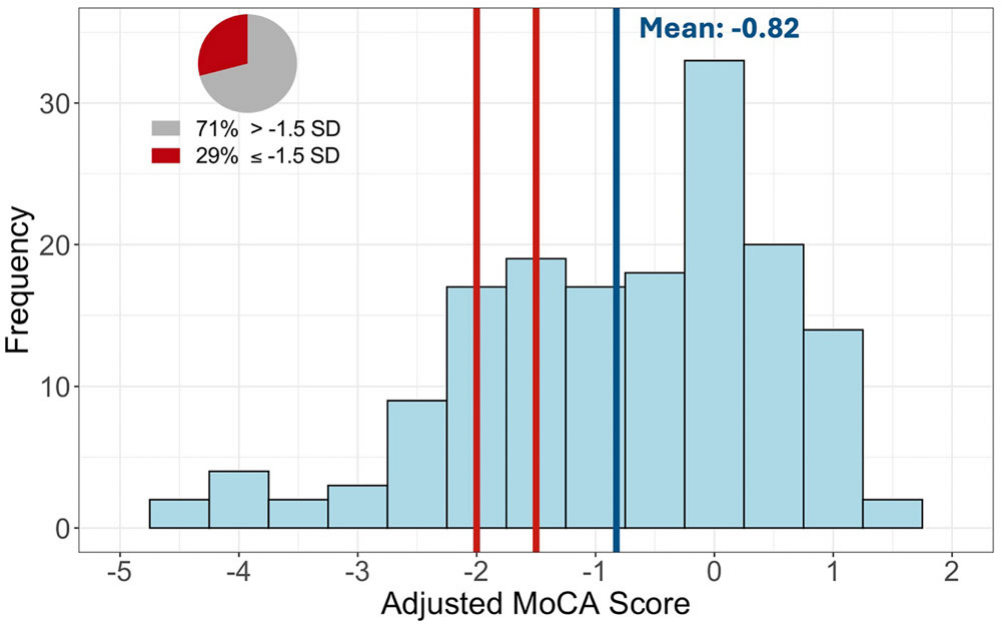
Distribution of NACC‐adjusted MoCA scores within this community‐dwelling vascular cohort. Frequency plot showing the distribution of age‐, sex‐, and education‐adjusted MoCA scores based on NACC normative data within our vascular cohort of 162 individuals. The blue line represents the mean normative MoCA score of the cohort. The red lines represent the –1.5 SD and –2 SD cutoffs, representing cognitive impairment compatible with MCI and dementia. Approximately 70% are below the normative population mean of zero; 30% of the cohort falls below the 1.5 SD threshold; 18.5% of the cohort falls below 2 SD. MCI, mild cognitive impairment; MoCA, Montreal Cognitive Assessment; NACC, National Alzheimer's Coordinating Center; SD, standard deviation.

**FIGURE 2:**
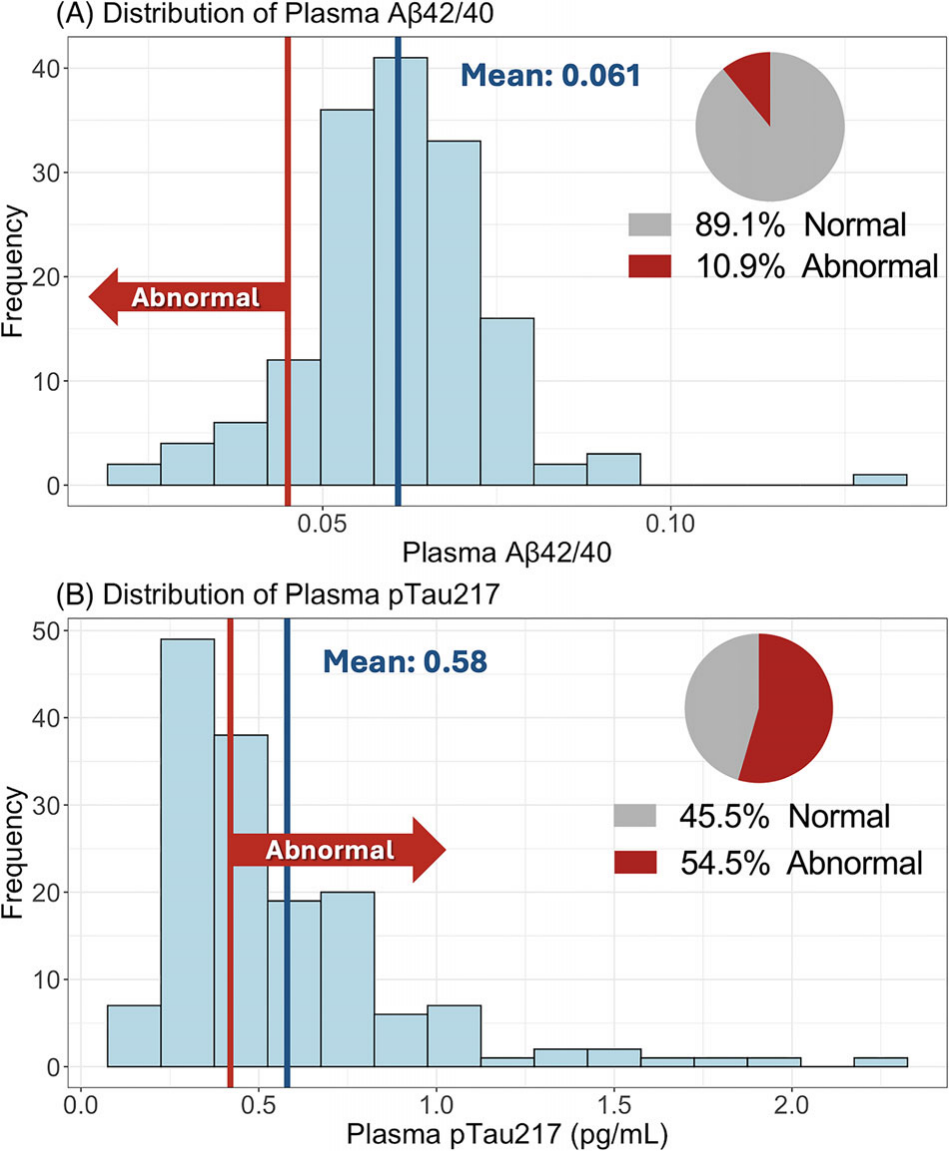
Distribution of plasma p‐tau217 and Aβ42/40 in this community‐dwelling vascular cohort. Frequency plots showing the distribution of (A) plasma Aβ42/40 and (B) p‐tau217 within the vascular cohort. The blue lines represent the mean plasma Aβ42/40 and p‐tau217 levels within the cohort. The red lines represent previously published cutoffs based on PET positivity. Applying these cutoffs, 10.9% and 54.5% of the cohort had abnormal levels of plasma Aβ42/40 and p‐tau217, respectively. Six participants were missing p‐tau217 and Aβ42/40 and were excluded from this analysis. One outlier was removed from the p‐tau217 frequency plot for data visualization purposes (p‐tau217 = 4.59) but was included in all statistical analyses. Aβ, amyloid beta; PET, positron emission tomography; p‐tau, phosphorylated tau.

**FIGURE 3:**
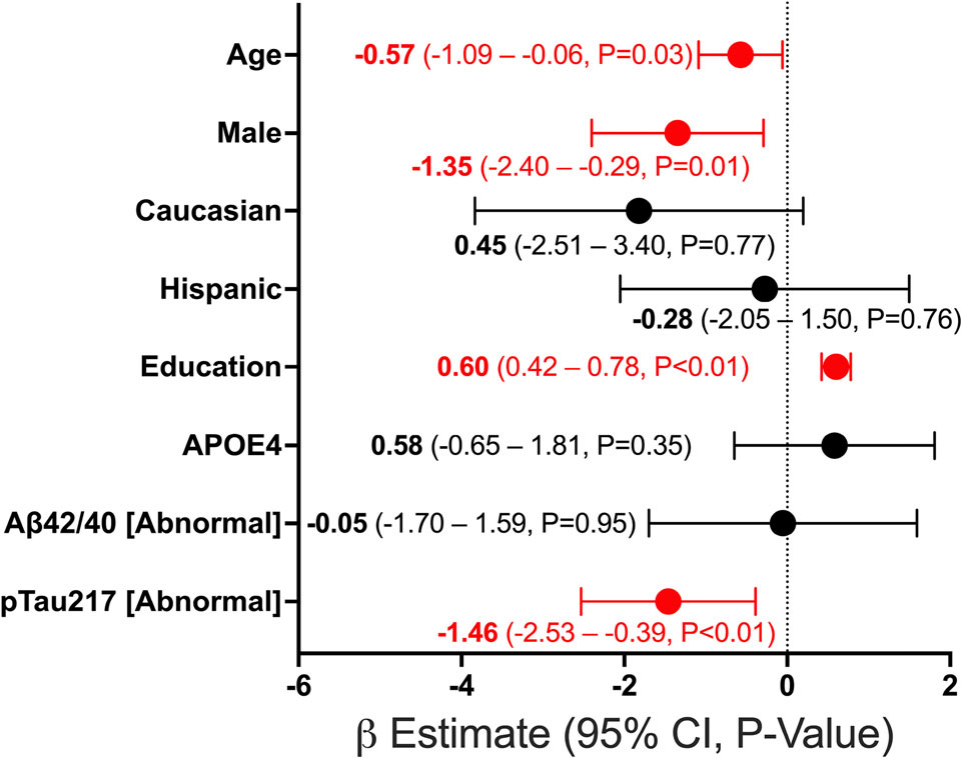
Plasma p‐tau217, but not Aβ42/40, is associated with worse cognitive performance in this community‐dwelling vascular cohort. A, Forest plot showing a significant relationship between plasma p‐tau217 and MoCA scores after adjusting for age (years), sex, race and ethnicity, education years, *APOE* ε4, and plasma Aβ2/40 using a multivariate linear regression model. No significant effect was observed for Aβ42/40. Each dot represents the beta estimates, with error bars representing the 95% confidence interval. The beta estimates, 95% confidence intervals, and *p* values are transcribed; *p* < 0.05 was considered statistically significant. Aβ, amyloid beta; *APOE*, apolipoprotein E; p‐tau, phosphorylated tau.

**FIGURE 4:**
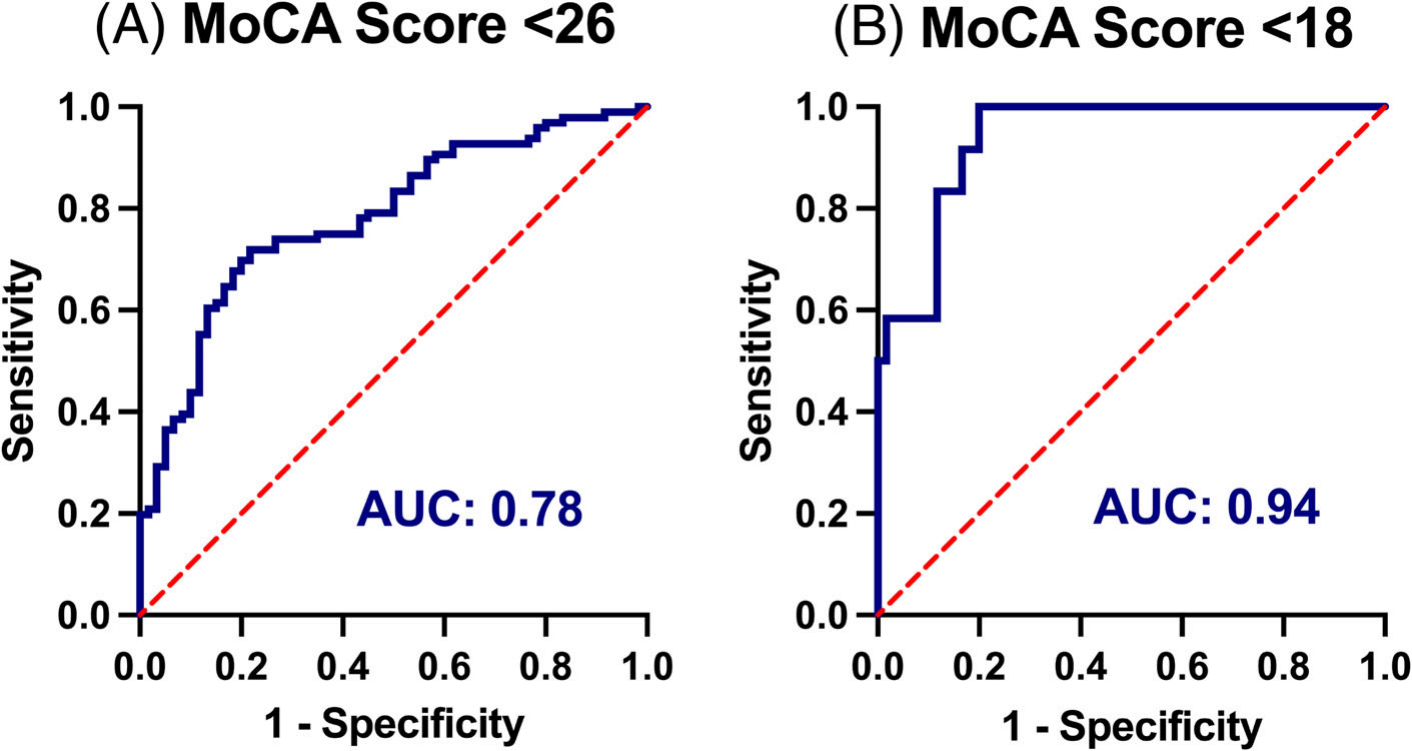
Plasma p‐tau217 detects cognitive impairment with high accuracy. Receiver operating characteristic (ROC) plots were generated by partitioning study participants into normal (MoCA ≥ 26), mild (MoCA 18–25), and moderate (MoCA ≤ 17) cognitive status: (A) shows normal versus mild + moderate (*n* = 156) and (B) shows normal versus moderate (*n* = 72). p‐tau217 was used as a continuous variable. MoCA scores were adjusted for age, sex, education, and race and ethnicity. AUC, area under the curve; MoCA, Montreal Cognitive Assessment; p‐tau, phosphorylated tau.

The study cohort was 43.2% female, 95.1% White, 9.9% Hispanic, and 71% had at least a high school education. The general and clinical demographics are summarized in Table 1. As expected, the cohort contained high rates of cardiovascular comorbidities. Forty‐two (26.9%) were heterozygous for the *APOE* ε4 allele and none were homozygous; 3 participants had an *APOE* ε2/ε4 genotype and the remaining 39 participants had an *APOE* ε3/ε4 genotype. Overall, this vascular cohort had lower scores on the MoCA than expected for individuals of the same age, sex, and education level (mean = −0.82, standard deviation [SD] = 1.35, Figure 1) based on the NACC UDS 3.0 normative sample.^33^ Sixty‐nine percent scored below an adjusted MoCA score of zero, demonstrating performance below the NACC‐adjusted normative mean (Figure 1). In addition, 29.0% had an adjusted MoCA score of ≥ 1.5 SD below the mean, indicating neurocognitive performance consistent with at least MCI; 18.5% had an adjusted MoCA score ≥ 2.0 SD below the mean, which is compatible with dementia (Figure 1).^36^, ^37^ Within our cohort, 22 (13.6%) participants were below the age of 65 and these individuals had a similar degree of cognitive impairment compared to the older adults, with 27.2% scoring < 1.5 SD below the mean. Based on published cutoffs,^22^, ^32^ 11% and 55% of the cohort had plasma Aβ42/40 and p‐tau217 levels in agreement with amyloid PET positivity, respectively (*N* = 156, Figure 2). Abnormal levels of p‐tau217 (≥ 0.42 pg/mL) were significantly associated with worse raw MoCA scores (*β* = −2.20, 95% confidence interval [CI] −3.38 to −1.03, *p* < 0.01, Figure S2 in supporting information), even after adjusting for age, sex, race and ethnicity, education, *APOE* ε4, and Aβ42/40 (*β* = −1.46, 95% CI −2.53 to −0.39, *p* < 0.01). Aβ42/40 had no association with raw MoCA scores in unadjusted (*β* = 0.398, 95% CI −1.56 to 2.36, *p* = 0.68, Figure S2) or adjusted models (*β* = −0.05, 95% CI −1.70 to 1.59, *p* = 0.95, Figure 3). Furthermore, p‐tau217 exhibited high accuracy for detecting individuals with a MoCA < 26 (area under the curve [AUC] 0.78, 95% CI 0.71 to 0.86, *p* < 0.01) and < 18 (AUC 0.94, 95% CI 0.88 to 0.99, *p* < 0.01) in a ROC analysis adjusted for age, sex, education, and race and ethnicity (Figure 4).

**TABLE 1:** Summary of cohort demographics.

Characteristic	*N* = 162
Age	72.1 (7.4)
Female	70 (43.2)
Education years	
≤12	46 (28.4)
13–16	80 (49.4)
≥17	36 (22.2)
Mean (SD)	14.5 (2.9)
Race	
Asian	5 (3.1)
Black	1 (0.6)
White	154 (95.1)
Other	1 (0.6)
Not reported	1 (0.6)
Ethnicity	
Hispanic	16 (9.9)
Non‐Hispanic	141 (87.0)
Not reported	5 (3.1)
MoCA score	24.0 (3.9)
Range	12–30
p‐tau217 (pg/mL)	0.58 (0.47)^d^
Aβ42/40	0.061 (0.014)^d^
*APOE* ε4 carrier^*^	42 (26.9)^d^
Obesity^a^	43 (26.5)
Smoking^b^	91 (56.2)
Atrial fibrillation^b^	17 (10.5)
Hypertension^c^	105 (64.8)
Hyperlipidemia^c^	121 (74.7)
Diabetes^c^	43 (26.5)
aECAD^c^	103 (63.6)
Coronary artery disease^c^	60 (37.0)
Myocardial infarction^c^	16 (9.9)
Peripheral arterial disease^c^	31 (19.1)
Stroke^c^	27 (16.7)
Transient ischemic attack^c^	10 (6.2)
Chronic kidney disease^c^	11 (6.8)

*Note*: Continuous data are shown as mean (SD), while categorical data are shown as count (%).

Abbreviations: Aβ, amyloid beta; aECAD, asymptomatic extracranial carotid atherosclerotic disease; *APOE*, apolipoprotein E; MoCA, Montreal Cognitive Assessment; p‐tau, phosphorylated tau; SD, standard deviation.

^a^ Defined as body mass index ≥ 30.

^b^ Defined as current smoker or ≥ 10 pack‐year history.

^c^ Defined by the presence of a clinical diagnosis or taking associated medications.

^d^ Calculated based on *N* = 156 participants with plasma biomarkers and *APOE* genotypes available.

### 3.2 Secondary analysis

Because stroke is a known risk factor for cognitive impairment and dementia,^34^ we performed sensitivity analyses to account for its effects in our cohort. After excluding 27 participants with a past medical history of stroke, 65.9% had adjusted MoCA scores below zero. In addition, 25.2% and 13.3% scored ≤ 1.5 and 2.0 SD, respectively (Figure S3 in supporting information). Fifty percent of the remaining cohort had elevated p‐tau217. Interestingly, in participants with a distant history of stroke, 13 (48.1%) scored ≥ 1.5 SD below the mean, suggesting the possibility of residual cognitive deficits. Further, p‐tau217 remained significantly associated with lower MoCA scores in the full cohort after adjusting for stroke and other potential confounders (*β* = −1.30, 95% CI −2.37 to −0.22, *p* = 0.02, Figure S3). There remained no association observed between plasma Aβ42/40 and cognitive performance after adjusting for stroke (*β* = 0.12, 95% CI −1.52 to 1.77, *p* = 0.88, Figure S3).

In addition, CKD has been shown to increase p‐tau217 independent of AD pathology;^35^ therefore, sensitivity analyses were conducted to account for its potential effects in our cohort. After excluding 11 participants with a past medical history of CKD, 27.2% and 17.2% of the remaining cohort scored ≤ 1.5 and 2.0 SD, respectively, and 51% had elevated p‐tau217 levels. Further, p‐tau217 remained significantly associated with lower MoCA scores in the full cohort independent of CKD and other potential confounders (*β* = −1.26, 95% CI −2.35 to −0.17, *p* = 0.02, Figure S3), while no association was observed for Aβ42/40 (*β* = −0.12, 95% CI −1.76 to 1.52, *p* = 0.89, Figure S3).

## 4 DISCUSSION

In this prospective, cross‐sectional cohort involving community‐dwelling patients with asymptomatic vascular disease without diagnoses of cognitive dysfunction or dementia, we found high rates of cognitive impairment, with 29% of participants scoring ≥ 1.5 SD below the NACC‐adjusted normative mean on the MoCA. Additionally, 55% had elevated p‐tau217, the most reliable blood biomarker of AD; this degree of elevated p‐tau217 is greater than the 22% reported in a cohort recruited from the general population with similar age, sex, and *APOE* ε4 prevalence.^35^

All participants had primary care doctors and were additionally seen by at least one vascular specialist, yet none had prior clinical diagnoses of MCI or dementia, presumably from lack of evaluation of cognitive health. As expected, this cohort had a higher prevalence of vascular comorbidities compared to the general population.^38^, ^39^, ^40^, ^41^ *APOE* ε4 allele prevalence was 26.9% in this cohort (with no homozygotes), similar to the general US population.^42^ We found no effect of *APOE* ε4 status on cognitive performance or plasma biomarkers. Similarly, the Atherosclerosis Risk in Communities study found that the increased risk of AD associated with hypertension, diabetes, and smoking was independent of *APOE* ε4.^43^ Other work showed that vascular risk factors did not increase dementia risk in *APOE* ε4 carriers.^44^, ^45^ Another study determined that vascular risk factors increase risk for dementia to a larger extent in *APOE* ε4 non‐carriers.^46^ Altogether, these data suggest that some neurodegeneration associated with vascular disease may be distinct from *APOE* ε4‐related neurodegeneration.

Despite focusing on community‐dwelling patients without a clinical diagnosis of dementia or MCI, we found that roughly 70% of this cohort scored below the adjusted normative mean MoCA score; 29% had MoCA scores ≥ 1.5 SD below the mean, in agreement with MCI; and 18.5% scored ≥ 2.0 SD below the mean, compatible with dementia. Based on the NACC normative sample, accounting for differences in age, sex, and education, this cohort has greater cognitive impairment than would be expected in the general population (roughly 10%).^33^, ^47^ In a primary care cohort of 872 adults ≥ 55 years (mean age 66.8 years, 44.7% male), 10.3% had a MoCA score ≥ 1.5 SD below the mean adjusted for age and education (mean −0.12 ± 1.02 SD).^47^ In a cohort of patients without diagnosed MCI presenting for vascular surgery, 60.5% of the patients had a MoCA score ≤ 24, in agreement with MCI and similar to our unadjusted MoCA data (Figure S4 in supporting information).^48^ This study and related data from other vascular cohorts^49^ are limited because they are not adjusted for age, sex, or education, which can greatly affect neurocognitive scores and their interpretation. Additionally, these patients likely had increased stress by being tested prior to surgery, which may further confound results. However, the data are consistent with our findings of elevated levels of cognitive impairment in vascular patients beyond what would be expected in the general population.

Because of the high prevalence of neurocognitive deficits we found in this cohort, we sought to further understand the implications by quantifying plasma Aβ42/40 and p‐tau217, which can be elevated years or decades prior to clinical dementia and correlate well with PET and CSF‐based biomarkers.^20^, ^22^, ^50^, ^51^ The distribution of p‐tau217 was skewed right (above published cutoff values for positivity) with 55% of the cohort having high p‐tau217 values (Figure 2). This degree of elevated p‐tau217 is greater than what has been found in a cohort of similar age, sex, and *APOE* ε4 status recruited from the general population without a diagnosis of dementia (roughly 22%).^35^ It is also greater than the similarly aged Wisconsin Registry for Alzheimer's Prevention cohort that is enriched for participants with parents with dementia (39%).^22^ Although there may be relevant limitations (discussed below), these data indicate that AD pathology is highly prevalent in this vascular cohort.

In contrast to p‐tau217, only 11% of the cohort had abnormal Aβ42/40 values. This differential AD biomarker finding may be reflective of Aβ42/40 immunoassay drift, low fold change across the AD spectrum, peripheral sources of amyloid, or other limitations of Aβ42/40 immunoassays and not reflective of differential brain pathology. However, p‐tau217 more accurately predicts tau brain pathology compared to Aβ pathology^22^ and is highly associated with Braak staging,^22^ which has been observed to be a better predictor of cognitive decline than Aβ.^52^ Additionally, our finding appears unique to vascular patient populations because testing done in general populations has shown greater concordance between Aβ42/40 and p‐tau217 levels.^23^ These differences between p‐tau217 and Aβ42/40 are aligned with previous work defining connections between vascular pathways and tau pathology, but less so with Aβ.^53^, ^54^, ^55^ Although more investigation is needed to fully understand the meaning and implication, we hypothesize that tauopathy could be more relevant in vascular‐related neurodegeneration seen in AD.

In keeping with these differential biomarker findings, p‐tau217, but not Aβ42/40, significantly correlated with cognitive performance in this cohort after controlling for age, sex, education, and *APOE* ε4 status. Others have similarly found that plasma p‐tau217 has greater association with cognitive decline than plasma Aβ42/40, regardless of manufacturer.^23^ Additionally, ROC analysis demonstrated that p‐tau217 is accurate in discriminating cognitive impairment in this cohort, further supporting the potential utility of plasma p‐tau217 in this population.

Because stroke is a well‐established risk factor for dementia,^34^ we performed sensitivity analyses to explore its relevance. The adjusted mean MoCA score of the stroke‐excluded participants remained below the NACC normative cohort mean, and 25% of participants remained ≥ 1.5 SD below the normative mean. Nearly half of the participants with a distant history of stroke scored ≥ 1.5 SD below the mean, suggesting the possibility of residual cognitive deficits. Additionally, the relationship between p‐tau217 and MoCA scores was not affected by excluding participants with stroke. These analyses support extension of the study findings to vascular patients with and without a history of a stroke.

This study has some limitations. (1) Neurocognitive assessment using MoCA testing alone is insufficient to diagnose MCI or dementia. Despite this limitation, the MoCA was selected because it is a quick and sensitive screening tool, which makes it ideal for incorporation into the clinic. Future studies could include a more thorough evaluation of neurocognitive and functional status needed to diagnose dementia. (2) Plasma Aβ42/40 and p‐tau217 have limited evaluations in vascular populations. However, this is a key reason the current study is novel, and having neurocognitive data to correlate with blood biomarkers provides relevant insight to help interpretation. To address this limitation further, we evaluated outcomes adjusted for CKD and a history of myocardial infarction (MI), which are potential confounders for plasma p‐tau217 levels.^35^ In our cohort, 6.8% had prior diagnoses of CKD. When these participants were excluded, 51% of the cohort still had elevated p‐tau217 levels and 27.2% had normative MoCA scores consistent with cognitive impairment and dementia (Figure 4C). Further, p‐tau217 remains significantly associated with decreased MoCA scores independent of CKD (Figure S3). A history of MI (9.9%) had no effect on AD biomarkers in our cohort (data not shown). (3) Blood collection parameters can affect results, with published data demonstrating lithium heparin vacutainers have differential plasma AD biomarker levels (higher^56^ or lower^57^) compared to K2‐EDTA. This limitation is relevant for determining cut‐off values but is mitigated in our analyses because p‐tau217 predicts cognitive performance with high accuracy, corroborating the relevance of p‐tau217 in this population (yet does not for Aβ42/40). (4) There is no internal “non‐vascular” control group within our analyses. This limitation was addressed using NACC normative data for neurocognitive assessment and by comparison to relevant publications evaluating “non‐vascular” cohorts with MoCA or plasma AD biomarker data. Of note, NACC is a rigorous neurocognitive data repository used to compare cohorts across studies, and much of the AD biomarker evaluation in cited publications was performed using the same assays at the same facility (NCRAD) as our own. (5) This cohort has higher rates of aECAD compared to other vascular diseases, which could limit generalizability; however, cardiac disease, carotid artery disease, and most of the other vascular comorbidities observed in this cohort have independent associations with cognitive dysfunction, and the study is underpowered to look at individual effects of vascular comorbidities. (6) Life expectancy in vascular populations is relevant for dementia risk consideration and could not be fully defined in this cohort. This limitation was addressed by excluding participants with symptomatic heart disease, symptomatic carotid disease, heart failure, and/or non‐benign cancers, which we considered the most relevant life‐limiting diseases found in this population.

In conclusion, the quantity and severity of cognitive impairment and AD pathology, as assessed by the MoCA, plasma p‐tau217, and plasma Aβ42/40, are high and underappreciated in a community‐dwelling vascular cohort without a past medical history of cognitive dysfunction or dementia. Our results support further studies evaluating the benefit of brain health screening in vascular patients. The potential for brain health screening in vascular patients is particularly relevant as vascular diseases represent modifiable contributors to early AD progression.

## CONFLICT OF INTEREST STATEMENT

J.L.D. is an inventor on patents or patent applications of Eli Lilly and Company relating to the assays, methods, reagents, and/or compositions of matter for p‐tau assays and Aβ targeting therapeutics. J.L.D. has served as a consultant or on advisory boards for Eisai, Abbvie, Genotix Biotechnologies Inc., Gates Ventures, Gate Neurosciences, Dolby Family Ventures, Karuna Therapeutics, AlzPath Inc., Cognito Therapeutics, Inc., Prevail Therapeutics, and received research support from ADx Neurosciences, Fujirebio, AlzPath Inc., Roche Diagnostics, and Eli Lilly and Company in the past 2 years. J.L.D. has received speaker fees from Eli Lilly and Company and LabCorp. J.L.D. is a founder and advisor for Monument Biosciences. J.L.D. has stock or stock options in Eli Lilly and Company, Genotix Biotechnologies, AlzPath Inc., and Monument Biosciences. E.M.R. is a co‐founder and advisor of ALZpath, and a compensated scientific advisor to Alzheon, Denali, Cognition Therapeutics, Enigma, Retromer Therapeutics, and Vaxxinity. While the Quanterix ALZpath assay was used to characterize pTau217 levels, he was not involved in the analysis of data. None of the other authors declare that they have conflicts of interest. Author disclosures are available in the supporting information.

## ETHICS STATEMENT

All experiments were conducted in accordance with the Declaration of Helsinki. All procedures are approved by the institutional review board at the University of Arizona (1606653257), and all participants, or their legal representatives, gave informed consent.

## CONSENT STATEMENT

All subjects or their legal representatives signed written informed consent.

## CONSENT FOR PUBLICATION

All authors listed on this manuscript are in agreement with the findings and their interpretation and provide consent for publication of this manuscript.

## CODE AVAILABILITY

All analytic software is publicly available as described in the methods section of this manuscript.

## Supporting information



Supporting information

Supporting Information

## Data Availability

The deidentified data used in the analyses for this study, including cognitive and plasma biomarker data, will be made available upon reasonable request to the corresponding author (C.W.) and receipt of a signed data access agreement form.

## References

[1] GBD 2019 Dementia Forecasting Collaborators . Estimation of the global prevalence of dementia in 2019 and forecasted prevalence in 2050: an analysis for the Global Burden of Disease Study 2019. Lancet Public Health. 2022;7(2):e105‐e125. doi:10.1016/S2468-2667(21)00249-8 34998485 PMC8810394

[2] Mattke S , Jun H , Chen E , Liu Y , Becker A , Wallick C . Expected and diagnosed rates of mild cognitive impairment and dementia in the U.S. Medicare population: observational analysis. Alzheimers Res Ther. 2023;15(1):128. doi:10.1186/s13195-023-01272-z 37481563 PMC10362635

[3] Amjad H , Roth DL , Sheehan OC , Lyketsos CG , Wolff JL , Samus QM . Underdiagnosis of dementia: an observational study of patterns in diagnosis and awareness in us older adults. J Gen Intern Med. 2018;33(7):1131‐1138. doi:10.1007/s11606-018-4377-y 29508259 PMC6025653

[4] van Dyck CH , Swanson CJ , Aisen P , et al. Lecanemab in early Alzheimer's disease. N Engl J Med. 2023;388(1):9‐21. doi:10.1056/NEJMoa2212948 36449413

[5] Lennon MJ , Lam BCP , Lipnicki DM , et al. Use of antihypertensives, blood pressure, and estimated risk of dementia in late life: an individual participant data meta‐analysis. JAMA Netw Open. 2023;6(9):e2333353. doi:10.1001/jamanetworkopen.2023.33353 37698858 PMC10498335

[6] Lee H , Lee S , Choi E , et al. Risk of dementia after smoking cessation in patients with newly diagnosed atrial fibrillation. JAMA Netw Open. 2022;5(6):e2217132. doi:10.1001/jamanetworkopen.2022.17132 35704317 PMC9201679

[7] Meng X , Yu J , Wang H , et al. Midlife vascular risk factors and the risk of Alzheimer's disease: a systematic review and meta‐analysis. J Alzheimers Dis. 2014;42(4):1295‐1310. doi:10.3233/jad-140954 25024338

[8] Gottesman RF , Schneider ALC , Zhou Y , et al. Association between midlife vascular risk factors and estimated brain amyloid deposition. JAMA. 2017;317(14):1443‐1450. doi:10.1001/jama.2017.3090 28399252 PMC5921896

[9] Kivipelto M , Helkala EL , Laakso MP , et al. Midlife vascular risk factors and Alzheimer's disease in later life: longitudinal, population based study. BMJ. 2001;322(7300):1447‐1451. doi:10.1136/bmj.322.7300.1447 11408299 PMC32306

[10] Malik R , Georgakis MK , Neitzel J , et al. Midlife vascular risk factors and risk of incident dementia: longitudinal cohort and Mendelian randomization analyses in the UK Biobank. Alzheimers Dementia. 2021;17(9):1422‐1431. doi:10.1002/alz.12320 33749976

[11] Whitmer RA , Sidney S , Selby J , Johnston SC , Yaffe K . Midlife cardiovascular risk factors and risk of dementia in late life. Neurology. 2005;64(2):277‐281. doi:10.1212/01.WNL.0000149519.47454.F2 15668425

[12] Gottesman RF , Albert MS , Alonso A , et al. Associations between midlife vascular risk factors and 25‐year incident dementia in the atherosclerosis risk in communities (ARIC) cohort. JAMA Neurol. 2017;74(10):1246‐1254. doi:10.1001/jamaneurol.2017.1658 28783817 PMC5710244

[13] Tolppanen AM , Solomon A , Soininen H , Kivipelto M . Midlife vascular risk factors and Alzheimer's disease: evidence from epidemiological studies. J Alzheimers Dis. 2012;32(3):531‐540. doi:10.3233/jad-2012-120802 22842867

[14] Ridker PM ; JUPITER Study Group . Rosuvastatin in the primary prevention of cardiovascular disease among patients with low levels of low‐density lipoprotein cholesterol and elevated high‐sensitivity c‐reactive protein. Circulation. 2003;108(19):2292‐2297. doi:10.1161/01.CIR.0000100688.17280.E6 14609996

[15] The Diabetes Prevention Program Research Group . The Diabetes Prevention Program. Design and methods for a clinical trial in the prevention of type 2 diabetes. Diabetes Care. 1999;22:623‐634. doi:10.2337/diacare.22.4.623 10189543 PMC1351026

[16] Grundy SM , Stone NJ , Bailey AL , et al. 2018 AHA/ACC/AACVPR/AAPA/ABC/ACPM/ADA/AGS/APhA/ASPC/NLA/PCNA Guideline on the management of blood cholesterol: a report of the American College of Cardiology/American Heart Association Task Force on clinical practice guidelines. Circulation. 2019;139(25):e1082‐e1143. doi:10.1161/CIR.0000000000000625 30586774 PMC7403606

[17] Whelton PK , Carey RM , Aronow WS , et al. 2017 ACC/AHA/AAPA/ABC/ACPM/AGS/APhA/ASH/ASPC/NMA/PCNA Guideline for the prevention, detection, evaluation, and management of high blood pressure in adults: a report of the American College of Cardiology/American Heart Association Task Force on clinical practice guidelines. Hypertension. 2018;71(6):e13‐e115. doi:10.1161/HYP.0000000000000065 29133356

[18] Brott TG , Halperin JL , Abbara S , et al. 2011 ASA/ACCF/AHA/AANN/AANS/ACR/ASNR/CNS/SAIP/SCAI/SIR/SNIS/SVM/SVS Guideline on the management of patients with extracranial carotid and vertebral artery disease: executive summary. Circulation. 2011;124(4):489‐532. doi:10.1161/CIR.0b013e31820d8d78 21282505

[19] Mistur R , Mosconi L , Santi SD , et al. Current challenges for the early detection of Alzheimer's disease: brain imaging and CSF studies. J Clin Neurol. 2009;5(4):153‐166. doi:10.3988/jcn.2009.5.4.153 20076796 PMC2806537

[20] Li Y , Yen D , Hendrix RD , et al. Timing of biomarker changes in sporadic Alzheimer's disease in estimated years from symptom onset. Ann Neurol. 2024;95(5):951‐965. doi:10.1002/ana.26891 38400792 PMC11060905

[21] Palmqvist S , Tideman P , Cullen N , et al. Prediction of future Alzheimer's disease dementia using plasma phospho‐tau combined with other accessible measures. Nat Med. 2021;27(6):1034‐1042. doi:10.1038/s41591-021-01348-z 34031605

[22] Ashton NJ , Brum WS , Di Molfetta G , et al. Diagnostic accuracy of a plasma phosphorylated tau 217 immunoassay for Alzheimer disease pathology. JAMA Neurol. 2024;81(3):255‐263. doi:10.1001/jamaneurol.2023.5319 38252443 PMC10804282

[23] Schindler SE , Petersen KK , Saef B , et al. Head‐to‐head comparison of leading blood tests for Alzheimer's disease pathology. Alzheimers Dementia. 2024;20(11):8074‐8096. doi:10.1002/alz.14315 PMC11567821 39394841

[24] Anastasi F , Fernández‐Lebrero A , Ashton NJ , et al. A head‐to‐head comparison of plasma biomarkers to detect Alzheimer's disease in a memory clinic. Alzheimers Dement. 2025;21(2):e14609. doi:10.1002/alz.14609 39998475 PMC11852974

[25] Palmqvist S , Janelidze S , Quiroz YT , et al. Discriminative accuracy of plasma phospho‐tau217 for Alzheimer disease vs other neurodegenerative disorders. JAMA. 2020;324(8):772‐781. doi:10.1001/jama.2020.12134 32722745 PMC7388060

[26] Riedhammer C , Halbritter D , Weissert R . Peripheral blood mononuclear cells: isolation, freezing, thawing, and culture. Methods Mol Biol. 2016;1304:53‐61. doi:10.1007/7651_2014_99 25092056

[27] Corrada MM , Hayden KM , Paganini‐Hill A , et al. Age of onset of hypertension and risk of dementia in the oldest‐old: the 90+ study. Alzheimers Dement. 2017;13(2):103‐110. doi:10.1016/j.jalz.2016.09.007 28108119 PMC5318224

[28] AlMansoori ME , Jemimah S , Abuhantash F , AlShehhi A . Predicting early Alzheimer's with blood biomarkers and clinical features. Sci Rep. 2024;14(1):6039. doi:10.1038/s41598-024-56489-1 38472245 PMC10933308

[29] Ho P , Yu WH , Tee BL , et al. Asian Cohort for Alzheimer's Disease (ACAD) pilot study on genetic and non‐genetic risk factors for Alzheimer's disease among Asian Americans and Canadians. Alzheimers Dement. 2024;20(3):2058‐2071. doi:10.1002/alz.13611 38215053 PMC10984480

[30] Rajbanshi B , Prufer Q C Araujo I , VandeVrede L , et al. Clinical and neuropathological associations of plasma Aβ(42)/Aβ(40), p‐tau217 and neurofilament light in sporadic frontotemporal dementia spectrum disorders. Alzheimers Dement. 2025;17(1):e70078. doi:10.1002/dad2.70078 PMC11780117 39886325

[31] Milà‐Alomà M , Tosun D , Schindler SE , et al. Timing of changes in Alzheimer's disease plasma biomarkers as assessed by amyloid and tau PET clocks. Ann Neurol. 2025. doi:10.1002/ana.27285 PMC12335434 40539416

[32] Colmant L , Boyer E , Gerard T , et al. Definition of a threshold for the plasma Aβ42/Aβ40 ratio measured by single‐molecule array to predict the amyloid status of individuals without dementia. Int J Mol Sci. 2024;25(2):1173. 38256246 10.3390/ijms25021173 PMC10816992

[33] Weintraub S , Besser L , Dodge HH , et al. Version 3 of the Alzheimer Disease Centers' Neuropsychological test battery in the Uniform Data Set (UDS). Alzheimer Dis Assoc Disord. 2018;32(1):10‐17. doi:10.1097/wad.0000000000000223 29240561 PMC5821520

[34] Pendlebury ST , Rothwell PM ; Oxford Vascular Study . Incidence and prevalence of dementia associated with transient ischaemic attack and stroke: analysis of the population‐based Oxford Vascular Study. Lancet Neurol. 2019;18(3):248‐258. doi:10.1016/s1474-4422(18)30442-3 30784556 PMC6390174

[35] Mielke MM , Dage JL , Frank RD , et al. Performance of plasma phosphorylated tau 181 and 217 in the community. Nat Med. 2022;28(7):1398‐1405. doi:10.1038/s41591-022-01822-2 35618838 PMC9329262

[36] Bradfield NI . Mild cognitive impairment: diagnosis and subtypes. Clin EEG Neurosci. 2023;54(1):4‐11. doi:10.1177/15500594211042708 34549629

[37] Pugh EA , Kemp EC , van Dyck CH , Mecca AP , Sharp ES ; Alzheimer's Disease Neuroimaging Initiative . Effects of normative adjustments to the Montreal cognitive assessment. Am J Geriatr Psychiatry. 2018;26(12):1258‐1267. doi:10.1016/j.jagp.2018.09.009 30314940 PMC6779033

[38] de Weerd M , Greving JP , Hedblad B , et al. Prevalence of asymptomatic carotid artery stenosis in the general population. Stroke. 2010;41(6):1294‐1297. doi:10.1161/STROKEAHA.110.581058 20431077 PMC4254855

[39] Meza R , Cao P , Jeon J , Warner KE , Levy DT . Trends in US Adult Smoking Prevalence, 2011 to 2022. JAMA Health Forum. 2023;4(12):e234213‐e234213. doi:10.1001/jamahealthforum.2023.4213 38038988 PMC10692849

[40] Karr S . Epidemiology and management of hyperlipidemia. Am J Manag Care. 2017;23(Suppl.9):S139‐s148. 28978219

[41] Shammas NW . Epidemiology, classification, and modifiable risk factors of peripheral arterial disease. Vasc Health Risk Manag. 2007;3(2):229‐234. doi:10.2147/vhrm.2007.3.2.229 17580733 PMC1994028

[42] Corbo RM , Scacchi R . Apolipoprotein E (APOE) allele distribution in the world. Is APOE*4 a ‘thrifty’ allele?. Ann Hum Genet. 1999;63(Pt 4):301‐310. doi:10.1046/j.1469-1809.1999.6340301.x 10738542

[43] Gottesman RF , Albert MS , Alonso A , et al. Associations between midlife vascular risk factors and 25‐year incident dementia in the atherosclerosis risk in communities (ARIC) cohort. JAMA Neurol. 2017;74(10):1246‐1254. doi:10.1001/jamaneurol.2017.1658 28783817 PMC5710244

[44] Kivipelto M , Helkala E , Laakso MP , et al. Apolipoprotein E epsilon4 allele, elevated midlife total cholesterol level, and high midlife systolic blood pressure are independent risk factors for late‐life Alzheimer disease. Ann Intern Med. 2002;137(3):149‐155. doi:10.7326/0003-4819-137-3-200208060-00006 12160362

[45] Shinohara M , Tashiro Y , Suzuki K , Fukumori A , Bu G , Sato N . Interaction between APOE genotype and diabetes in cognitive decline. Alzheimers Dement. 2020;12(1):e12006. doi:10.1002/dad2.12006 PMC7085280 32211501

[46] Smith JR , Pike JR , Gottesman RF , et al. Contribution of modifiable midlife and late‐life vascular risk factors to incident dementia. JAMA Neurology. 2025;82:644‐654. doi:10.1001/jamaneurol.2025.1495 40455489 PMC12131178

[47] Federman AD , Becker JH , Mindt MR , Cho D , Curtis L , Wisnivesky J . Rates of undiagnosed cognitive impairment and performance on the montreal cognitive assessment among older adults in primary care. J Gen Intern Med. 2023;38(11):2511‐2518. doi:10.1007/s11606-023-08102-w 36814049 PMC10465418

[48] Partridge JS , Dhesi JK , Cross JD , et al. The prevalence and impact of undiagnosed cognitive impairment in older vascular surgical patients. J Vasc Surg. 2014;60(4):1002‐1011.e3. doi:10.1016/j.jvs.2014.04.041 25017513

[49] Houghton JSM , Nickinson ATO , Bridgwood B , et al. Prevalence of cognitive impairment in individuals with vascular surgical pathology: a systematic review and meta‐analysis. Eur J Vasc Endovasc Surg. 2021;61(4):664‐674. doi:10.1016/j.ejvs.2020.12.016 33573912

[50] Janelidze S , Barthélemy NR , Salvadó G , et al. Plasma phosphorylated tau 217 and aβ42/40 to predict early brain aβ accumulation in people without cognitive impairment. JAMA Neurol. 2024;81(9):947‐957. doi:10.1001/jamaneurol.2024.2619 39068669 PMC11284634

[51] Barthélemy NR , Salvadó G , Schindler SE , et al. Highly accurate blood test for Alzheimer's disease is similar or superior to clinical cerebrospinal fluid tests. Nat Med. 2024;30(4):1085‐1095. doi:10.1038/s41591-024-02869-z 38382645 PMC11031399

[52] Nelson PT , Alafuzoff I , Bigio EH , et al. Correlation of Alzheimer disease neuropathologic changes with cognitive status: a review of the literature. J Neuropathol Exp Neurol. 2012;71(5):362‐381. doi:10.1097/NEN.0b013e31825018f7 22487856 PMC3560290

[53] Arias JC , Edwards M , Vitali F , Beach TG , Serrano GE , Weinkauf CC . Extracranial carotid atherosclerosis is associated with increased neurofibrillary tangle accumulation. J Vasc Surg. 2022;75:223‐228. doi:10.1016/j.jvs.2021.07.238 34478810 PMC8976507

[54] Albrecht D , Isenberg AL , Stradford J , et al. Associations between vascular function and tau PET are associated with global cognition and amyloid. J Neurosci. 2020;40(44):8573‐8586. doi:10.1523/jneurosci.1230-20.2020 33046556 PMC7605425

[55] Bennett RE , Robbins AB , Hu M , et al. Tau induces blood vessel abnormalities and angiogenesis‐related gene expression in P301L transgenic mice and human Alzheimer's disease. Proc Natl Acad Sci U S A 2018;115(6):E1289‐E1298. doi:10.1073/pnas.1710329115 29358399 PMC5819390

[56] Ashton NJ , Suárez‐Calvet M , Karikari TK , et al. Effects of pre‐analytical procedures on blood biomarkers for Alzheimer's pathophysiology, glial activation, and neurodegeneration. Alzheimers Dement. 2021;13(1):e12168. doi:10.1002/dad2.12168 PMC8171159 34124336

[57] Gouda M , Antwi‐Berko D , van Leeuwenstijn MSSA , et al. Plasma phosphorylated tau 217 levels are highly stable under common pre‐analytical sample handling procedures. Alzheimers Dementia. 2023;19(S14):e078393. doi:10.1002/alz.078393

