# Supplementary material for: Cognitive impairment and p‐tau217 are high in a vascular patient cohort
Source: Alzheimers Dement. 2025 Aug 7;21(8):e70565. doi: 10.1002/alz.70565 (PMC12329572; doi:10.1002/alz.70565)
Supplement: Supplementary file 2 — Supporting Information [file ALZ-21-e70565-s002.docx]

**Cognitive Impairment and pTau-217 are High in a Community-dwelling Vascular Disease Cohort: Supplemental Material**

**Supplemental Figure 1.** Plasma pTau217 and Aβ42/40 are stable in relation to length of time spent in storage at -80ºC. **(Page 2)**

**Supplemental Figure 2:** Forest plot depicting *univariate* relationships of plasma biomarkers with MoCA scores. **(Page 3)**

**Supplemental Figure 3.** Sensitivity analysis accounting for the effects of stroke and chronic kidney disease (CKD). **(Page 4)**

**Supplemental Figure 4:** Distribution of raw MoCA scores within this community-dwelling vascular patient cohort. **(Page 5)**

**
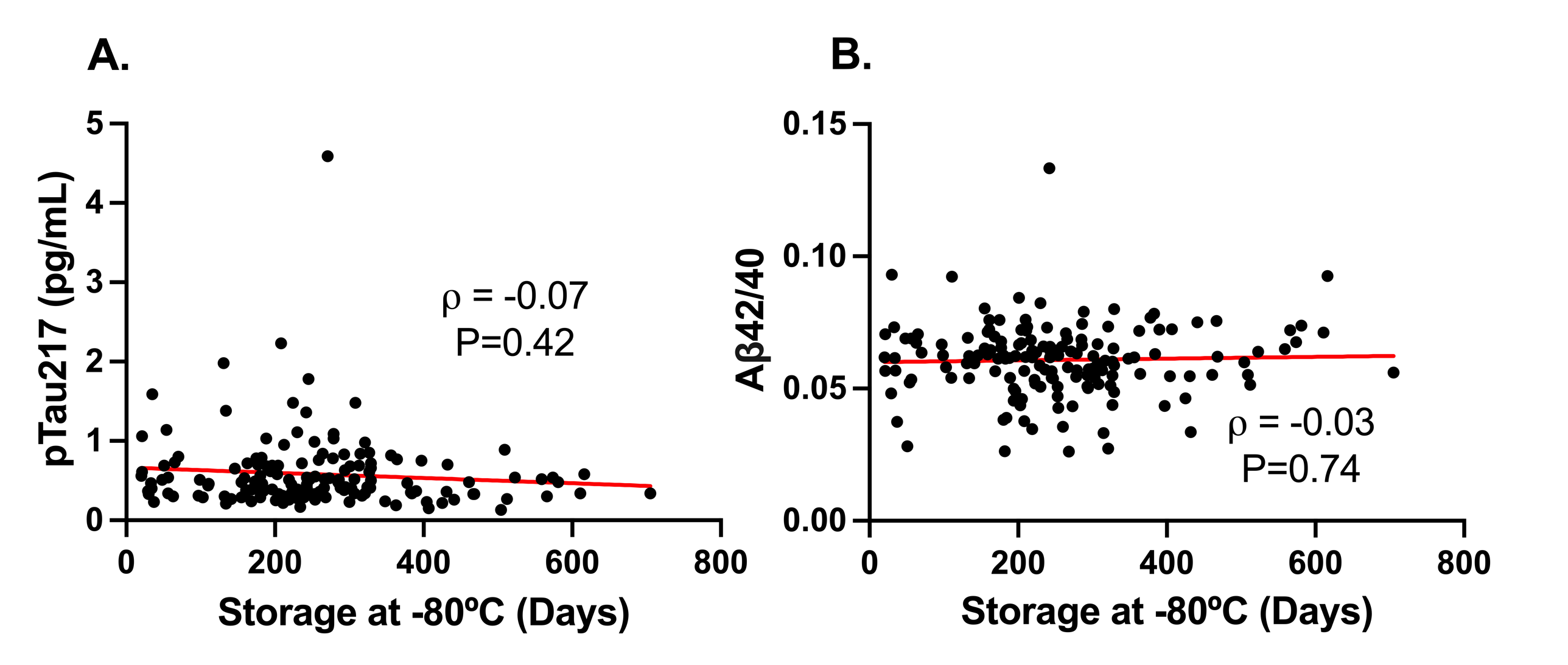
**

**Supplemental Figure 1. Plasma pTau217 and Aβ42/40** **are stable in relation to length of time spent in storage at -80ºC.** *Scatterplots showing no correlation between* ***A)*** *plasma pTau217 and* ***B)*** *plasma Aβ2/40 with the time spent in storage at -80ºC prior to running the assay in 156 participants (Spearman’s Correlation). 6 participants were missing pTau-217 and Aβ42/40 and were excluded from this analysis. The best-fit regression line is shown, and each dot represents a single participant. A P-value of less than 0.05 was considered statistically significant.*


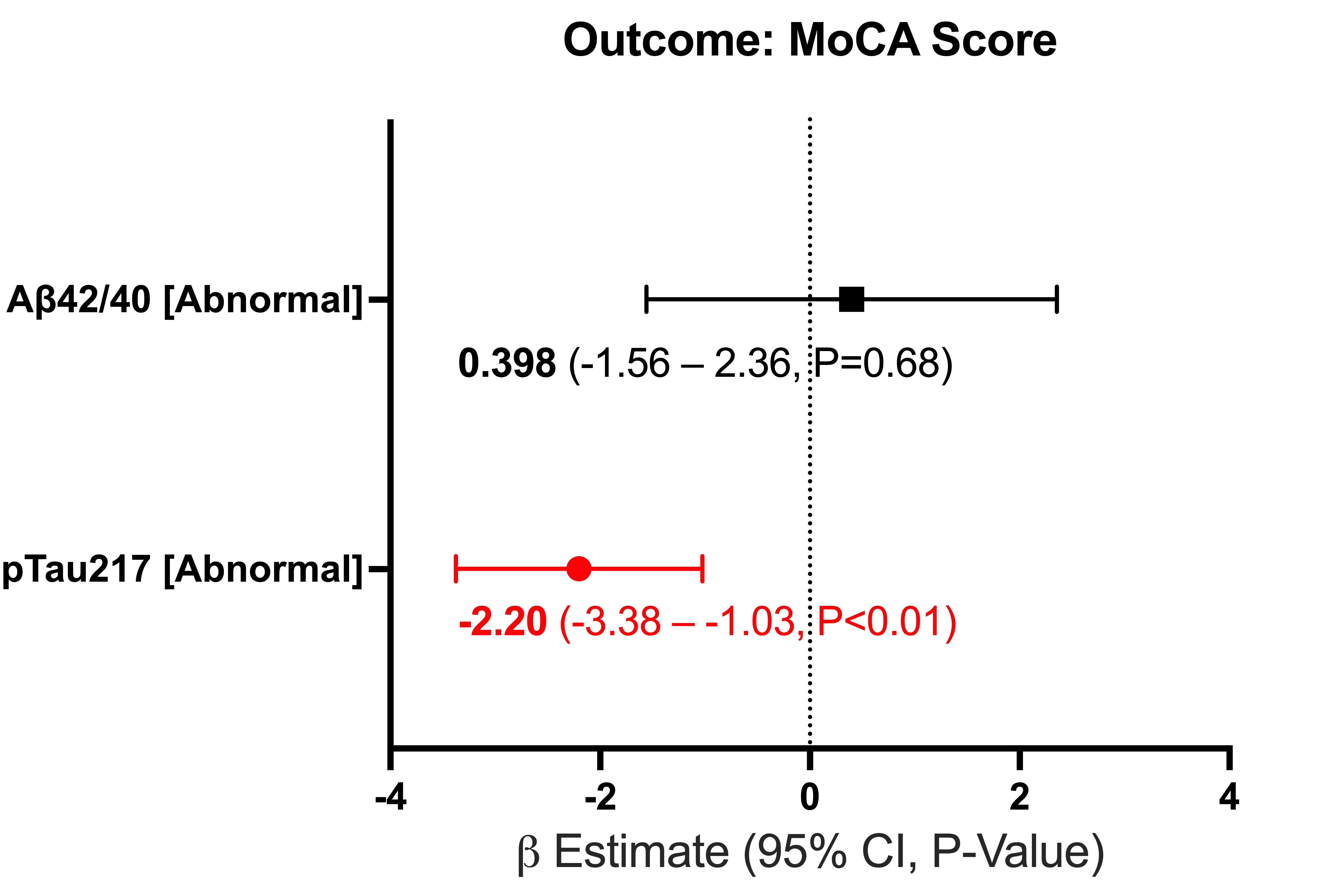


**Supplemental Figure 2.** **Forest plot depicting *univariate* relationships of plasma biomarkers with MoCA scores.** *The square and dot represent the beta-estimates of each univariate model, with error bars representing the 95% confidence interval. The beta estimates, 95% confidence intervals, and P-Values are transcribed; a P<0.05 was considered statistically significant.*

**
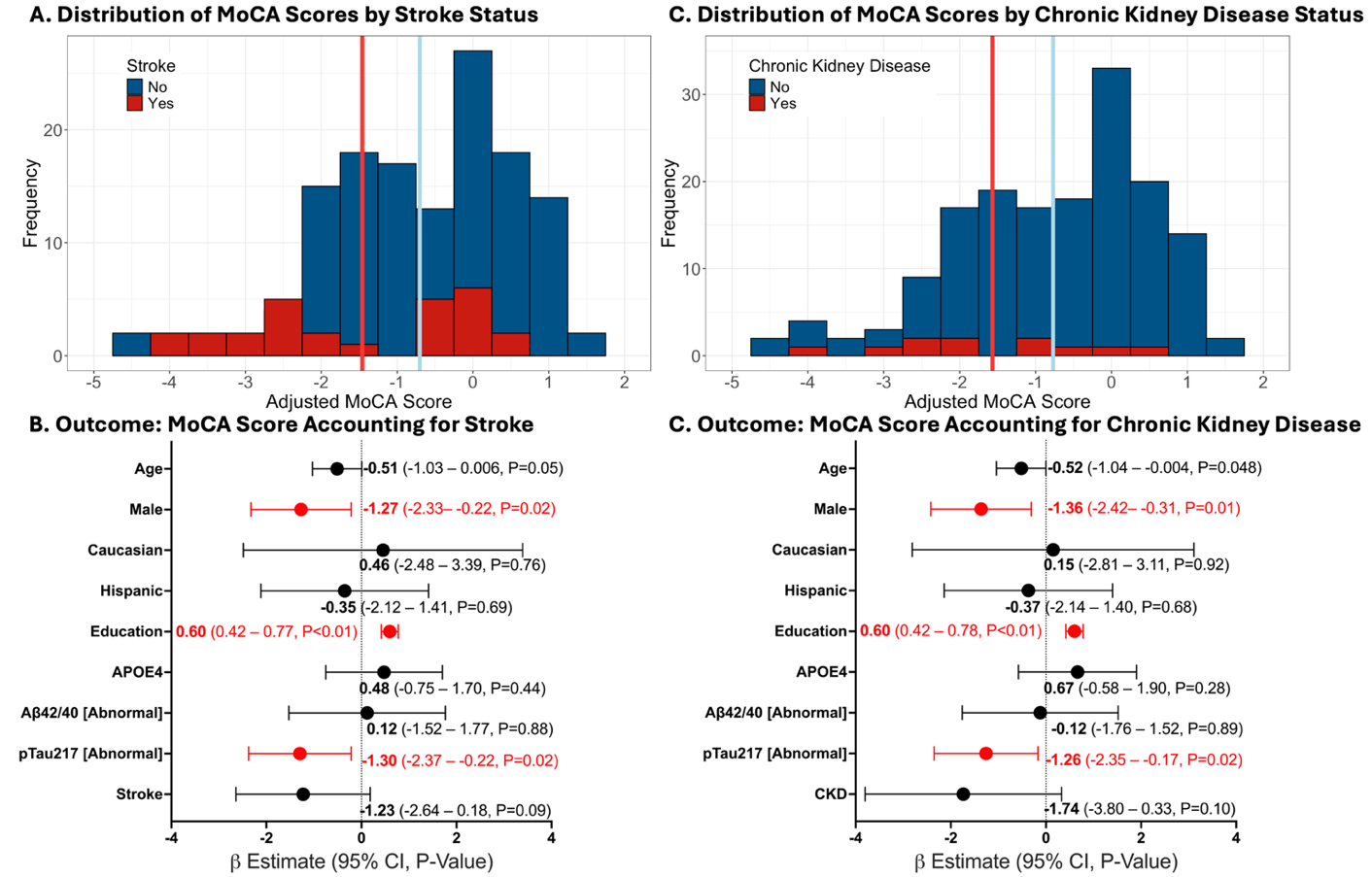
**

**Supplemental Figure 3. Sensitivity analysis accounting for the effects of stroke and chronic kidney disease (CKD)*.*** *Frequency plots are shown depicting the distribution of age, sex, and education adjusted MoCA scores within our vascular cohort of 162 individuals stratified by past medical history* *of* ***A) stroke and C) CKD****. The light red lines represent the mean normative MoCA scores within participants with stroke (-1.46) or CKD (-1.53). The light blue lines represent the mean normative MoCA scores within participants without stroke (-0.70) or CKD (-0.77).* ***B and D)*** *Forest plots showing a significant relationship between pTau-217 and MoCA scores after adjusting for* ***B) stroke and D) CKD*** *in the presence of other potential confounders within this vascular cohort. Each dot represents the beta-estimates, with error bars representing the 95% confidence interval. The beta estimates, 95% confidence intervals, and P-Values are transcribed; a P<0.05 was considered statistically significant.*


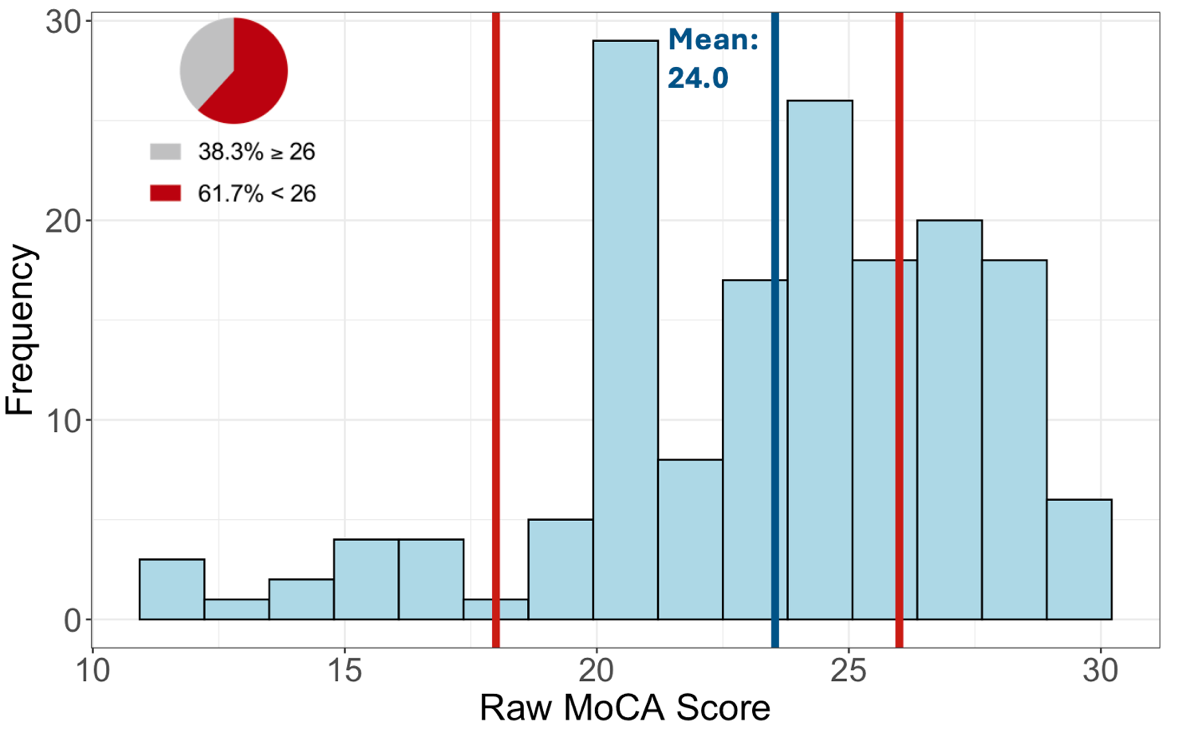


**Supplemental Figure 4. Distribution of raw MoCA scores within this community-dwelling vascular patient cohort.** *Frequency plot showing the distribution of raw MoCA scores within our vascular cohort of 162 individuals. The blue line represents the mean MoCA score of the cohort. The red lines represent the MoCA cutoffs of 26 and 18, representing cognitive impairment in agreement with MCI and dementia. 61.7% of the cohort scored below 26, while 8.6% of the cohort fell below 18.*
